# The effect of an on-site trauma surgeon during resuscitations of severely injured patients

**DOI:** 10.1186/s12873-022-00724-3

**Published:** 2022-09-28

**Authors:** Oscar E. C. van Maarseveen, Wietske H. W. Ham, Loek P. H. Leenen

**Affiliations:** 1 Department of Trauma Surgery, University Medical Center Utrecht, Heidelberglaan 100, 3584 CX Utrecht, The Netherlands; 2 Emergency Department, University Medical Center Utrecht, Heidelberglaan 100, 3584 CX Utrecht, The Netherlands; 3 University of Applied Science Utrecht, Institute of Nursing Studies, Utrecht, The Netherlands

**Keywords:** Resuscitation, Trauma team, Composition, Leadership, Experience

## Abstract

**Background:**

Although the timely involvement of trauma surgeons is widely accepted as standard care in a trauma center, there is an ongoing debate regarding the value of an on-site attending trauma surgeon compared to an on-call trauma surgeon. The aim of this study was to evaluate the effect of introducing an on-site trauma surgeons and the effect of their presence on the adherence to Advanced Trauma Life Support (ATLS) related tasks and resuscitation pace in the trauma bay.

**Methods:**

The resuscitations of severely injured (ISS > 15) trauma patients 1 month before and 1 month after the introduction of an on-site trauma surgeon were assessed using video analysis. The primary outcome was total resuscitation time. Second, time from trauma bay admission until tasks were performed, and ATLS adherence were assessed.

**Results:**

Fifty-eight videos of resuscitations have been analyzed. After the introduction of an on-site trauma surgeon, the mean total resuscitation time was 259 seconds shorter (*p* = 0.03) and seven ATLS related tasks (breathing assessment, first and second IV access, EKG monitoring and abdominal, pelvic, and long bone examination; were performed significantly earlier during trauma resuscitation (*p* ≤ 0.05). Further, we found a significant enhancement to the adherence of six ATLS related tasks (Airway assessment, application of a rigid collar, IV access; EKG monitoring, log roll, and pronouncing results of arterial blood gas analysis; *p*-value ≤0.05).

**Conclusion:**

Having a trauma surgeon on-site during trauma resuscitations of severely injured patients resulted in improved processes in the trauma bay. This demonstrates the need of direct involvement of trauma surgeons in institutions treating severely injured patients.

## Background

High quality trauma care delivery necessitates a strategy that combines a systematic approach, with clinical judgment and effective coordination. The introduction of trauma systems, including a well-structured resuscitation, has significantly improved the outcome of patients who have suffered severe injuries [1]. One of the pillars of a coordinated early resuscitation strategy has been the formation of in-hospital trauma teams [2–4]. A trauma team consists of multiple (para) medical members of various disciplines and the aim of the trauma team is to identify life-threatening injuries and administer prompt resuscitations and stabilization.

The trauma team leader is responsible for well-coordinated resuscitation and decision making during the resuscitation [5–7]. The value of a trauma surgeon during the resuscitation of severely injured patients has been well established and the timely involvement of trauma surgeons is recommended in the American College of Surgeons’ trauma guidelines [8]. However, the concept of ‘timely’ is ambiguous, and as such, there is an ongoing debate regarding the value an in on-site trauma surgeon during trauma resuscitations. Several studies have found that the introduction on-site attending trauma surgeons, compared to on-call trauma surgeons, enhance efficiency of the trauma resuscitation, such as faster decisions, fewer errors, shorter time to disposition, and shorter hospital stays [9–12]. This demonstrates that the early involvement of experienced trauma team leaders will enhance the quality of initial trauma care.

However, studies that use video analysis to evaluate processes in the trauma bay after the introduction of an in-hospital attending trauma surgeons are lacking. Previous mentioned studies evaluated process efficiency by retrospectively analyzing available data, such as data on emergency department or in-hospital lead times. This study aims to evaluate the direct effect of the introduction of a physical attendance of a trauma surgeon (on-site) during the initial trauma resuscitation of severely injured patients on process efficiency (resuscitation time) and quality in the trauma bay.

## Method

### Design, sample and outcomes

This study utilized a ‘before and after’ study design using video analysis to evaluate the effects of introducing an attending trauma surgeon. All videos of severely injured patients resuscitated by a trauma team between the period of June 1st, 2013 and July 31st, 2013 were assessed a researcher Severely injured patients were defined as having trauma injuries that resulted in an Injury Severity Score (ISS) of > 15.The primary outcome was (A) total resuscitation time which was defined as time from the patient’s arrival in the trauma bay until patient left the resuscitation bay. Secondary outcomes were (B) the time to perform tasks related to the Advanced Trauma Life Support (ATLS) guidelines – which was defined as the time between patient arrival and the completion of ATLS task assessment – as well as (C) the adherence which was defined as the completion of the 26 tasks as prescribed in the ATLS guidelines. Resuscitations were assessed on twenty-six tasks predefined ATLS related tasks. (Table 1). The Dutch National Trauma Database (DNTD) regional data was used to describe patient demographics (age, gender) and explanatory variables (ISS, trauma mechanism).

**Table 1:** Baseline characteristics and total resuscitation time

	Before transition (*n* = 28)	After transition (*n* = 30)	*p*-value
Gender; male (%)	17 (61)	15 (50%)	0.290
ISS median (IQR)	25 (20–34)	20 (17–25)	0.01*
Age median (IQR)	47 (24–52)	48 (25–67)	0.98
Trauma mechanisme; blunt (%)	28 (100)	30 (100%)	N.A.
Total resuscitation time; seconds (SD)	1404 (264)	1145 (357)	0.03*

### Location

The University Medical Center of Utrecht is a 1000-bed tertiary care center accredited by the Joint Commission International (JCI). Our hospital meets all the American College of Surgeons’ Committee on Trauma’s (ACS-COT) requirements for a level-1 trauma center, with the exception of an onsite CT scan, which is located outside the emergency department instead of in the resuscitation bay.

### Intervention

On 1 July 2013, attending trauma surgeons transitioned from an out-of-hospital (OH) on-call schedule to an in-house (IH) schedule. Prior to this transition, trauma surgeons were available for on-call consultation and a senior resident in general surgery served as house officer. After the transition, there was a continuous presence of a trauma surgeon and a resident in general surgery (varying from junior to senior). As a result, instead of only a surgical resident prior to the transition, both a trauma surgeon and a surgical resident were present from the start of every trauma resuscitation after the transition, irrespective of the (expected) injury severity of the trauma patient. There were no other differences in trauma team composition and detailed description of the trauma team composition could be found in the article of Kreb et al. [13].

### Statistics

The continuous data with a normal distribution are expressed as means and SDs (total resuscitation time), whereas the non-normally distributed data are presented as medians with IQRs (ISS, Age, time from admission to ATLS related task performed). Student t-tests were used to compare parametric continuous data from before and after the transition and Mann-Whitney U tests were used for non-parametric continuous data. The χ2 test and Fisher’s exact test were used to compare categorical variables (patient characteristics: sex, type of trauma; ATLS adherence). All statistical analyses were performed using SPSS (IBM Corp. Released 2011. IBM SPSS Statistics for Windows, Version 20.0. Armonk, NY: IBM Corp.) A *p*-value of less than 0.05 was deemed statistically significant.

## Results

### Population

During the period of the study, fifty-eight videos of resuscitations of severely injured patients were captured and analyzed, and of the fifty-eight videos, twenty-six resuscitations were captured before the transition and thirty resuscitations after the transition. The demographics and baseline clinical characteristics before and after the transition was comparable, except for the median ISS (before transition 25 [IQR 20–34] vs after transition 20 [IQR 17–25] (*p* = 0.01) (Table 1).

### Time management

The total resuscitation time was significantly shorter after the transition with a time of 1404 seconds [SD 264] vs 1145 seconds [SD 357], *p* = 0.03 (Table 1). The median time of seven of the twenty-six ATLS related tasks were significantly lower after the transition (Breathing assessment 285 seconds [IQR 225–365] vs 192 seconds [171–245], *p* < 0.01; First IV access 566 seconds [546–870] vs 296 seconds [247–348], *p* = 0.02; Second IV access 820 seconds [485–994] vs 404 seconds [338–480], *p* < 0.01; EKG monitoring 232 seconds [212–324] vs 200 seconds [180–218], *p* = 0.02; Pelvic examination 310 seconds [259–393] vs 213 seconds [194–358], *p* < 0.01; Abdominal examination 307 seconds [242–387] vs 212 seconds [191–368], *p* = 0.01 and Long Bone examination 321 seconds [251–403] vs 221 seconds [207–379], *p* = 0.01). None of the ATLS related tasks were performed faster before the transition (Table 2).

**Table 2:** ATLS adherence and time management

	Adherence (percentage)			Time in seconds (IQR)		
	Before transition	After transition	*p*-value	Before transition	After transition	*p*-value
Handover information	28/28 (100%)	30/30 (100%)	–	122 (109–168)	122 (108–142)	0.55
Airway Assessment	19/28 (68%)	25/30 (83%)	0.03*	186 (123–255)	172 (149–200)	0.97
Breathing assessment	28/28 (100%)	30/30 (100%)	–	285 (225–362)	192 (171–245)	< 0.01*
Oxygen administration	23/28 (82%)	27/30 (90%)	0.47	220 (113–266)*	193 (143–606)*	1.00
Pulse oximeter	28/28 (100%)	30/30 (100%)	–	227 (152–289)	174 (150–251)	0.07
IV access 1	28/28 (100%)	30/30 (100%)	–	566 (546–870)*	296 (247–348)	0.02*
IV access 2	13/28 (68%)	23/30 (77%)	0.03*	820 (485–994)	404 (338–480)	< 0.01*
Withdrawal of blood samples	28/28 (100%)	30/30 (100%)	–	484 (411–599)	507 (380–562)	0.62
pronouncing results of arterial blood gas analysis	15/28 (54%)	24/30 (80%)	< 0.01*	793 (732–1025)	867 (756–984)	0.62
EKG monitor	16/28 (57%)	25/30 (83%)	0.04*	232 (212–324)	200 (180–218)	0.02*
Order of blood products	3/28 (11%)	0/30 (0%)	0.11	361 (81–511)*	–	NA
arrival of blood products	0/28 (0%)	0/30 (0%)	–	–	–	NA
Blood pressure and heart rate	28/28 (100%)	30/30 (100%)	–	279 (204–339)	217 (184–298)	0.13
Pelvic examination	28/28 (100%)	30/30 (100%)	–	310 (259–393)	213 (194–358)	< 0.01*
Abdominal examination	22/28 (79%)	27/30 (90%)	0.29	307 (242–387)	212 (191–368)	0.01*
Long bone examination	24/28 (86%)	26/30 (87%)	1.0	321 (251–403)	221 (207–379)	0.01*
Pupil examination	18/28 (62%)	26/30 (87%)	0.07	317 (265–420)	338 (278–450)	0.58
Neurological examination	12/28 (43%)	20/30 (67%)	0.11	537 (496–658)	422 (364–627)	0.28
Log Roll	3/28 (11%)	12/30 (40%)	0.02*	697 (572–721)	460 (369–598)	0.09
rectal examination	2/28 (7%)	6/28 (21%)	0.26	785 (408–948)*	461 (389–711)	0.18
Temperature measurement	0/28 (0%)	1/30 (3%)	1.0	–	1851**	NA
Warm Blankets	27/28 (96%)	30/30 (100%)	0.48	286 (201–332)	253–213-295)	0.62
Intubation	12/28 (43%)	9/30 (30%)	0.41	528 (413–765)*	434 (382–538)	0.08
Spine Control	22/28 (79%)	29/30 (97%)	0.05*	–	178 (128–252)*	NA
Chest Tube	3/28 (11%)	3/30 (10%)	1.0	1377 (639–1800)	859 (764–1472)*	0.827
Urinary catheter	14/28 (50%)	22/30 (73%)	*0.*10	894 (737–1201)	980 (664–1345)	0.893

^* ^= signifficantly different

### ATLS adherence

Six of the twenty-six ATLS related tasks regarding the primary survey were performed significantly more often after the transition when compared to before the transition (second IV access 68% vs 77%, *p* = 0.03; EKG monitoring 57% vs 83%, *p* = 0.04, pronouncing results of arterial blood gas analysis 54% vs 80%, *p* < 0.01; Log Roll 11% vs 40%, *p* = 0.02, Airway assessment 68% vs 83%, *p* = 0.02) and spine control 79% vs 97%, *p* = 0.05). None of the assessment tasks or interventions were performed more frequently after the transition (Table 2).

## Discussion

We found a significant acceleration of the resuscitation process in the trauma bay for severely injured patients directly after the introduction of trauma surgeons physically attending the trauma resuscitations. Total resuscitation time was significantly shorter and seven ATLS related tasks (breathing assessment, first and second IV access, EKG monitoring and abdominal, pelvic and long bone examination) were performed significantly earlier during trauma resuscitation. Furthermore, we found a significant enhancement of ATLS adherence of six tasks (airway assessment, application of a rigid collar, IV access; EKG monitoring, log roll and pronouncing results of arterial blood gas analysis).

These findings are consistent with those of a prior study in which we assessed the impact of introducing a 24-hour in-house attending trauma surgeon. In-hospital lead times of severely injured patients (ISS > 24) of patients before (*n* = 214) and after (*n* = 392) were compared and there was a significant decrease in the length of stay in the emergency department. (median 2.7 h (IQR 1.6–4.0) vs 2.1 h (1.5–3.7) *p* < 0.01) There was also a significant decrease in time from the ED (Emergency Department) to the intensive care unit (ICU) for patients directly transferred to the ICU (median time of 1.4 h (IQR 1.1–2.5) vs 1.2 h (IQR 1.0–1.6) (*p* < 0.01) and almost doubling of the percentage of patients who reached the operation room within 30 minutes (4.8% vs 8.0%) [14]. Moreover, a recent systematic of de la Mar et al., [15] revealed that ten out of sixteen included studies found at least one process related outcome to be improved after the implementing an in-house trauma surgeon attendance strategy. Furthermore, 7490 severely injured patients were included in a meta-analysis from eight different studies and severely injured patients treated by on-site surgeons had a significantly lower mortality rate when compared to trauma surgeons on call (risk ratio 0.86, 95% confidence interval 0.78 to 0.95; *P* < 0.01).

The improved time management and ATLS adherence are most likely a direct result of the addition of experience. It is debatable what effect a four-minute reduction in resuscitation time (as roughly found in our study) would have on clinical outcomes of the patient. The impact of such a time reduction will largely depend on the population being studied. Till date there is still is limited objective data to support time targets from injury to definitive care and 4 min reduction won’t significantly improve the outcome for those who don’t have acute life-threatening injuries. However, there is almost no doubt that earlier interventions save lives in a subset of severely injured casualties. Among these injuries, acute hemorrhage remains the leading cause of preventable deaths [5]. Aside from reasoning the shorter resuscitation time as a result of an on-site trauma surgeon, the shorter resuscitation time and improved adherence to ATLS guidelines reflect improved management of severely injured patients. Management of severely injured patients entails more than just time management and protocol adherence. We believe that an attending (experienced) surgeon can adapt to the complex nature of resuscitation of severely injured patients better than surgical residents. .. Moreover, in the context of resuscitation of severely injured trauma patients, non-technical abilities such as leadership, decision-making, and situation awareness have been described as critical [5, 7], and given that non-technical abilities often increase with practice, it is reasonable to think that the presence of an attending surgeon during the trauma resuscitation of severely injured patients is required for optimal trauma care. This reasoning is also supported by research of other institutes such as Cole et al. [16] who found that diagnostic imaging and hemorrhage control were significantly more likely achieved during resuscitation led by a trauma surgeon compared to a resident. Moreover, even additional experience for trauma surgeons during their career may improve resuscitation outcomes. In the study of McKenney et all [17]. a correlation between time as faculty surgeon at a level one trauma center and mortality in the most severely injured patients (ISS > 35) was found. In addition, Hong et al. [18], found that patients suffering severe neurotrauma (GCS ≤8) had a higher change to survive when resuscitated by an experienced trauma team leader when compared to a less experienced trauma team leader (odds ratio (OR): 14.5, 95% CI 1.7–125.5, *p* = 0.015).

### Strengths and limitations

The risk for information bias was minimized by the fact that solely one trained person assessed video recordings. This risk was further minimized by the actual use of video recordings itself as video analysis of trauma resuscitations are a reliable and valid method to assess processes during trauma resuscitation. However, this study has also several limitations that should be considered. First, the Injury Severity Score (ISS) was higher before the transition. In another recent study performed at our institution, we found a negative correlation between resuscitation time and patient severity of injury, implying that more severely injured patients were resuscitated faster than less severely injured patients [19]. Moreover, Spanjersberg and colleagues [20] discovered comparable results, with resuscitation times of severely injured patients (Revised Trauma Score (RTS) of < 12) being shorter (35 minutes) than less severely injured patients (RTS 12; 46 minutes). This may imply that the influence of an on-site trauma surgeon during trauma resuscitation on resuscitation time may even be underestimated. According to prior studies, longer resuscitation times should have been expected for patients with less serious injuries. However, the introduction of a trauma surgeon on-site did even shorten the time needed to complete the resuscitations by more than 4 minutes. The study’s second limitation is the small number of reviewed cases, which increases the likelihood of randomness and prevents more advanced (statistical) analysis. However, this study contributes to the existing literature because it is the first to evaluate the introduction of an on-site trauma surgeon and through video analysis. As a result, the findings of this study indicate that an on-site trauma surgeon is very likely to improve the pace and ATLS adherence of trauma resuscitations. Larger studies evaluating the effect of an on-site trauma surgeon during trauma resuscitation on are unlikely until automatic computer analysis will become available. Such automated video analysis is already being used in professional sports [20]. A third limitation of this study was that ATLS adherence was measured based on a predefined list of ATLS related tasks. Strictly, not all tasks are always indicated for every case. For example, a second IV line for patient hemodynamically stable patient may be overtreatment. This may imply that the ATLS adherence is undervalued, as not all tasks are indicated during the individual cases. However, the videos of patients that are analyzed, are patient that are relatively severe injured (ISS > 15) and therefore, we believe that most of the predefined ATLS related tasks are mostly indicated.

## Conclusion

In conclusion, the addition of a trauma surgeon’s physical presence during trauma resuscitations of severely injured patients resulted in improved processes in the trauma bay and this demonstrates the need of direct involvement of trauma surgeons in institutions treating severely injured patients.

## Data Availability

The datasets generated and/or analysed during the current study are not publicly available due to privacy regulations, but are available from the corresponding author on reasonable request.

## Abbreviations

ACS-COT: The American College of Surgeons’ Committee on Trauma’s
ATLS: Advanced Trauma Life Support
CT: Computed Tomography
DNTD: The Dutch National Trauma Database
EKG: Electrocardiogram
GCS: Glasgow Coma Score
ICU: Intensive Care Unit
IH: In-Hospital
IQR: Inter Quartile Range
ISS: Injury Severity score
IV: Intravenous
JCI: Joint Commission international
OH: Out-of-Hospital
OR: Odds Ratio
RTS: Revised Trauma score
